# AI-derived CT morphometric phenotypes predict survival, functional decline, and surgical morbidity following curative-intent surgical sarcoma resection

**DOI:** 10.1186/s13018-026-06851-y

**Published:** 2026-04-15

**Authors:** Julian Kylies, Elias Brauneck, Dominik Bannier, Tobias Dust, Alonja Reiter, Anna Duprée, Jana Striefler, Karl-Heinz Frosch, Matthias Priemel

**Affiliations:** 1 Department of Trauma and Orthopedic Surgery, University Medical Center Hamburg-Eppendorf, Hamburg, Germany; 2 Department of General, Visceral and Thoracic Surgery, University Medical Center Hamburg-Eppendorf, Hamburg, Germany; 3 Department of Oncology, Hematology and Bone Marrow Transplantation With Section Pneumology, Hubertus Wald University Cancer Center, University Medical Center Hamburg-Eppendorf, Hamburg, Germany; 4 Department of Trauma Surgery, Orthopaedics and Sports Traumatology, BG Klinikum Hamburg, Hamburg, Germany

## Abstract

**Background:**

Outcomes after curative-intent sarcoma surgery vary substantially and are incompletely explained by tumor-centered factors alone. Although CT-based body composition metrics provide objective host-related information, most sarcoma studies rely on isolated parameters or binary sarcopenia definitions. AI-driven analytical approaches offer the opportunity to integrate multidimensional morphometric data into data-driven phenotypes that may better capture clinically relevant heterogeneity.

**Methods:**

In this retrospective cohort study, our institutional sarcoma database (n = 2667) was screened to identify patients with osteosarcoma, myxofibrosarcoma, liposarcoma, or chondrosarcoma who underwent curative-intent surgical resection and had a preoperative CT including mid-L3 vertebral level for morphometric analysis (final cohort n = 234). Skeletal muscle index (SMI), skeletal muscle density (SMD), and visceral adipose tissue area (VAT) were quantified from a single axial mid-L3 slice. Unsupervised k-means clustering of standardized SMI, SMD, and VAT identified AI-derived morphometric phenotypes. Outcomes included overall survival (OS), ECOG performance status at follow-up, surgical site infection (SSI requiring surgical revision), and length of hospital stay (LOS). Multivariable regression models evaluated independent associations between phenotypes and outcomes, adjusting for relevant clinical covariates.

**Results:**

Clustering identified four phenotypes: muscle-preserved (n = 88), myosteatotic (n = 62), sarcopenic (n = 56), and cachexia-like (n = 28). Morphometric profiles differed markedly: muscle-preserved (SMI 47.8 ± 6.3 cm^2^/m^2^; SMD 41.6 ± 6.4 HU; VAT 118 ± 56 cm^2^), myosteatotic (SMI 42.1 ± 5.9; SMD 28.9 ± 5.3; VAT 142 ± 62), sarcopenic (SMI 35.6 ± 4.8; SMD 34.0 ± 5.6; VAT 96 ± 48), and cachexia-like (SMI 31.2 ± 4.4; SMD 26.4 ± 4.9; VAT 64 ± 35). Median OS differed significantly across phenotypes (155 vs. 32 vs. 64 vs. 19 months; *p* < 0.0001). Postoperative functional status also worsened stepwise (median ECOG at follow-up: 1 ± 0.5 vs. 2.5 ± 1 vs. 3 ± 1 vs. 3 ± 0.5; *p* < 0.0001). In multivariable Cox regression, cachexia-like (HR 3.28, 95% CI 2.01–5.36; *p* < 0.001) and sarcopenic phenotypes (HR 1.89, 95% CI 1.26–2.83; *p* = 0.002) independently predicted mortality, whereas conventional sarcopenia did not. SSI rates increased across phenotypes (6.8% to 21.4%; *p* = 0.042), cachexia-like (HR 3.21, 95% CI 1.69–6.10; *p* < 0.001) and sarcopenic phenotypes (HR 2.08, 95% CI 1.17–3.70; *p* = 0.012) were independently associated with SSI. LOS was independently prolonged in sarcopenic (+ 3.4 days, *p* = 0.002) and cachexia-like patients (+ 6.2 days, *p* < 0.001).

**Conclusions:**

AI-derived CT morphometric phenotypes obtained from routine preoperative imaging identify distinct host profiles in sarcoma patients and independently predict survival, postoperative functional decline and postoperative morbidity beyond conventional CT-based sarcopenia assessments. Integrating morphometric phenotyping into preoperative assessment may support risk stratification, counseling, and targeted perioperative optimization in curative-intent sarcoma surgery.

**Supplementary Information:**

The online version contains supplementary material available at 10.1186/s13018-026-06851-y.

## Introduction

Sarcomas are a rare and heterogeneous group of malignant tumors of mesenchymal origin that frequently require complex surgical resection as the cornerstone of curative treatment [1–3]. Despite advances in multimodal therapy, outcomes after sarcoma surgery remain highly variable [1, 4–6]. Even among patients undergoing curative-intent resection, postoperative morbidity, functional decline, and long-term survival differ markedly and are not fully explained by tumor-centered factors such as histology, grade, or stage alone [7, 8]. This variability highlights the growing importance of host-related factors, including physiological reserve and systemic disease burden, in determining surgical risk and oncologic outcomes.

In recent years, CT-based body composition analysis has emerged as a powerful and readily available tool to quantify objective markers of patient fitness, including skeletal muscle mass, muscle quality, and adipose tissue distribution [9–12]. Numerous studies across oncologic and surgical disciplines have demonstrated associations between sarcopenia, myosteatosis, or visceral adiposity and adverse outcomes such as increased complications, prolonged hospitalization, and reduced survival [13–22]. In sarcoma patients, however, available data remain limited and are largely restricted to single-parameter approaches, most commonly binary definitions of sarcopenia. Such models fail to reflect the biological complexity of cancer-associated body composition changes and overlook the substantial heterogeneity among patients who may share similar muscle mass but differ profoundly in muscle quality and adipose tissue profiles.

Importantly, skeletal muscle quantity, muscle radiodensity, and visceral fat represent interrelated yet distinct dimensions of host physiology. Their combined assessment may better capture the continuum from preserved physiology to myosteatosis, sarcopenia, and cancer cachexia [23]. Unsupervised machine-learning approaches enable the integration of these parameters into data-driven morphometric phenotypes without reliance on arbitrary thresholds. Recent work in oncologic surgery has shown that such phenotyping strategies can identify biologically and clinically meaningful patient subgroups that outperform conventional sarcopenia in prognostic stratification [24–26].

Therefore, the aim of the present study was to apply AI-based unsupervised clustering of preoperative CT-derived skeletal muscle index, skeletal muscle density, and visceral adipose tissue to identify morphometric phenotypes in patients undergoing curative intent sarcoma resection. We further sought to investigate whether these phenotypes are associated with baseline clinical characteristics and independently predict overall survival (OS), postoperative functional decline, surgical site infection (SSI), and length of hospital stay (LOS). We hypothesized that CT-based morphometric phenotypes would stratify sarcoma patients into distinct host profiles with differential oncologic and surgical risk, beyond conventional sarcopenia and established clinical predictors.

## Materials and methods

### Study design and patient selection

This retrospective cohort study was approved by the local institutional ethics committee and conducted in accordance with the Declaration of Helsinki. Owing to the retrospective and fully anonymized nature of the data, the requirement for written informed consent was waived (ID: 2025-300576-WF).

**Fig. 1:**
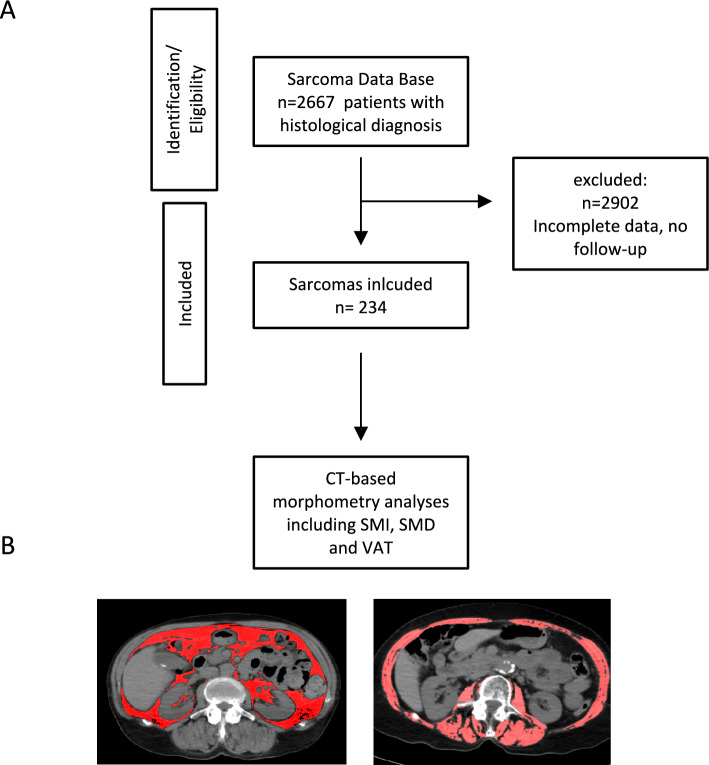
Study design. **A** Illustrated is the study design and patients selection process. Additionally, examples of CT-based morphometry are shown (**B**)

The institutional sarcoma database was systematically screened to identify all patients treated at our tertiary musculoskeletal oncology center. Between 2010 and 2024, a total of 2,667 patients with histologically confirmed sarcoma were registered. After application of all inclusion and exclusion criteria, a final cohort of 234 patients was included in the present analysis (Fig. 1). Patients were eligible if they (1) had a histologically confirmed sarcoma diagnosis, (2) underwent curative-intent surgical resection of the primary tumor, and (3) had a preoperative CT scan including the mid-L3 vertebral level available for morphometric analysis.

Patients were excluded if CT image quality was insufficient for morphometric analysis due to motion artifacts, severe metal-induced scatter, or incomplete abdominal coverage, if imaging did not adhere to institutional acquisition standards, or if relevant clinical or outcome data were incomplete. To minimize confounding, patients with concomitant active malignancies or systemic diseases known to independently influence skeletal muscle composition (e.g., neuromuscular disorders, inflammatory myopathies, or non-oncologic cachexia) were excluded.

Clinical and demographic data were extracted from the institutional electronic medical record system and anonymized prior to analysis. Collected variables included age, sex, sarcoma subtype, tumor location, tumor grade, preoperative ECOG performance status, comorbidity burden (Charlson Comorbidity Index), neoadjuvant and adjuvant treatment (chemotherapy and/or radiotherapy), and surgical characteristics.

Postoperative outcomes included OS, SSI, LOS, TNM status at diagnosis and ECOG performance status at follow-up. SSI was defined as any postoperative wound infection requiring surgical revision of the operative site. LOS was defined as the number of days from the index operation to hospital discharge. OS was calculated from the date of surgery to death from any cause or last follow-up.

### CT acquisition and morphometric analysis

All CT scans were acquired using standardized institutional imaging protocols on identical or cross-calibrated multidetector CT systems to ensure comparability of attenuation values. Axial images were reconstructed using routine soft-tissue kernels with consistent slice thickness and exported in DICOM format for further analysis.

Morphometric analysis was performed using Fiji/ImageJ software (Version 2.3.0/1.53q; Max Planck Institute of Molecular Cell Biology and Genetics, Dresden, Germany). For each patient, a single axial slice at the mid-L3 vertebral level was selected, representing a validated anatomical landmark for body composition assessment and a surrogate of whole-body skeletal muscle mass.

Using a semi-automated segmentation workflow, regions of interest were manually delineated for total skeletal musculature. Muscle tissue was isolated using standardized Hounsfield unit thresholds from − 29 to + 150 HU. Cross-sectional skeletal muscle area was normalized to height squared to calculate the skeletal muscle index (SMI, cm^2^/m^2^). Muscle quality was assessed by calculating the mean skeletal muscle radiation attenuation (SMD, HU) as a surrogate of intramuscular fat infiltration. Visceral adipose tissue area (VAT, cm^2^) was segmented at the same level using established adipose tissue thresholds as previously described. All segmentations were performed by trained investigators blinded to clinical characteristics and outcomes.

Preoperative sarcopenia was defined according to previously validated sex-specific cut-off values (SMI < 52.4 cm^2^/m^2^ for males and < 38.5 cm^2^/m^2^ for females). Conventional sarcopenia was used exclusively for comparative analyses and was not employed in phenotype generation [19, 22].

### AI-derived morphometric phenotyping

Unsupervised clustering was performed to identify data-driven morphometric phenotypes based exclusively on preoperative body composition parameters. The clustering input matrix consisted of skeletal muscle index (SMI, cm^2^/m^2^), skeletal muscle density (SMD, HU), and visceral adipose tissue area (VAT, cm^2^), with one observation per patient. Prior to clustering, all morphometric variables were inspected for distributional plausibility and outliers. Features were then z-score standardized (mean = 0, standard deviation = 1) to ensure equal weighting and to eliminate scale-dependent effects. Clustering was conducted in Python (Python Software Foundation, version 3.11) using the scikit-learn library. A k-means algorithm with Euclidean distance was applied, using k-means centroid initialization, 100 random initializations, and a maximum of 300 iterations per run to ensure convergence and minimize local minima effects. The optimal number of clusters (k) was explored iteratively for k = 2–8 using within-cluster sum of squares (elbow method), average silhouette coefficients, and visual inspection of principal component projections and heatmap representations to assess cluster separation, internal consistency, and biological plausibility. Based on convergence of these criteria, a four-cluster solution demonstrated the optimal balance between statistical robustness and clinical interpretability and was therefore selected. Cluster robustness was evaluated by repeating clustering across multiple random seeds and assessing stability of cluster membership and centroid profiles. Resulting clusters were characterized by their standardized centroid profiles and subsequently interpreted as muscle-preserved, myosteatotic, sarcopenic, and cachexia-like phenotypes.

Importantly, clustering was fully unsupervised and outcome-agnostic. No clinical variables, oncologic characteristics, or outcome data were incorporated into phenotype generation.

### Statistical analysis

Continuous variables are presented as mean ± standard deviation or median with interquartile range, as appropriate. Categorical variables are reported as counts and percentages. Intergroup comparisons were performed using one-way ANOVA or Kruskal–Wallis tests for continuous variables and chi-square or Fisher’s exact tests for categorical variables.

OS was analyzed using Kaplan–Meier methods and compared between phenotypes using the log-rank test. Multivariable Cox proportional hazards regression was performed to assess the independent association between morphometric phenotypes and mortality, adjusting for age, sex, sarcoma subtype, tumor grade, preoperative ECOG performance status, Charlson Comorbidity Index, neoadjuvant therapy and TNM status. Neoadjuvant therapy was defined as any oncologic treatment administered prior to surgical resection and included both systemic therapy (chemotherapy) and local therapy (radiotherapy). For primary analyses, neoadjuvant therapy was included as a binary variable (yes/no).

Postoperative SSI was analyzed using multivariable logistic regression. LOS was assessed using multivariable linear regression. Morbidity models were adjusted for age, preoperative ECOG performance status, comorbidity burden, tumor grade, and perioperative oncologic treatment.

Hazard ratios (HR), odds ratios (OR), beta coefficients, and corresponding 95% confidence intervals (CI) were reported. Statistical significance was defined as *p* < 0.05. All analyses were conducted using SPSS (IBM Corp., Armonk, NY) and Python-based statistical libraries. Graphical representations were prepared using GraphPad Prism 10.

To assess the potential confounding effect of treatment-related alterations in body composition, a sensitivity analysis was performed after exclusion of all patients who received neoadjuvant systemic therapy. All regression models were repeated using identical model specifications in this restricted cohort.

## Results

### Patient characteristics at baseline

A total of 234 patients undergoing curative-intent sarcoma resection were included and stratified into four morphometric phenotypes: muscle-preserved (n = 88), myosteatotic (n = 62), sarcopenic (n = 56), and cachexia-like (n = 28) (Table 1).

**Table 1:** Baseline patient characteristics and Ai-derived body composition types

Variable	Muscle-preserved (n = 88)	Myosteatotic (n = 62)	Sarcopenic (n = 56)	Cachexia-like (n = 28)	p-value
Female sex, n (%)	42 (47.7)	30 (48.4)	26 (46.4)	12 (42.9)	0.93
Age, years (mean ± SD)	56.9 ± 15.2	63.8 ± 13.8	67.4 ± 12.9	72.1 ± 11.6	< 0.001
High grade (G2–3), n (%)	44 (50.0)	39 (62.9)	40 (71.4)	24 (85.7)	< 0.001
Systemic therapy, n (%)	24 (27.3)	24 (38.7)	28 (50.0)	18 (64.3)	< 0.001
Radiotherapy, n (%)	12 (13.6)	12 (19.4)	16 (28.6)	10 (35.7)	0.006
Local recurrence, n (%)	14 (15.9)	16 (25.8)	18 (32.1)	12 (42.9)	0.003
Metastatic progression, n (%)	10 (11.4)	10 (16.1)	12 (21.4)	10 (35.7)	0.011
ECOG ≥ 2, n (%)	18 (20.5)	20 (32.3)	26 (46.4)	18 (64.3)	< 0.001
Charlson Comorbidity Index	4.2 ± 2.1	5.2 ± 2.3	6.0 ± 2.6	7.1 ± 2.9	< 0.001
SMI (cm^2^/m^2^)	47.8 ± 6.3	42.1 ± 5.9	35.6 ± 4.8	31.2 ± 4.4	< 0.001
SMD (HU)	41.6 ± 6.4	28.9 ± 5.3	34.0 ± 5.6	26.4 ± 4.9	< 0.001
VAT (cm^2^)	118 ± 56	142 ± 62	96 ± 48	64 ± 35	< 0.001
Surgical site infection, n (%)	6 (6.8)	7 (11.3)	8 (14.3)	6 (21.4)	0.042
Length of hospital stay, days	9.4 ± 4.3	11.2 ± 5.6	13.6 ± 6.8	17.8 ± 8.1	< 0.001
Median overall survival, months	155	32	64	19	< 0.001

Sex distribution was comparable across phenotypes, with no significant differences in the proportion of female patients (*p* = 0.93). In contrast, age differed significantly between groups, with a progressive increase from the muscle-preserved to the cachexia-like phenotype (56.9 ± 15.2 vs. 72.1 ± 11.6 years, *p* < 0.001). Histological diagnosis included osteosarcomas (n = 36), Liposarcomas (n = 64), Chondrosarcomas (n = 79) and Myxofibrosarcomas (n = 55). The selected histological subtypes (osteosarcoma, liposarcoma, myxofibrosarcoma, and chondrosarcoma) represent common bone and soft tissue sarcomas treated with curative-intent surgical resection at our institution. These entities were analyzed collectively to enable evaluation of host-related risk factors across a clinically relevant sarcoma population undergoing major oncologic surgery. The median follow-up for the overall cohort was 52 months (IQR 24–96 months).

More adverse oncologic characteristics were increasingly prevalent across deteriorating morphometric phenotypes. The proportion of high-grade tumors (G2–3) rose significantly from 50.0% in the muscle-preserved group to 85.7% in the cachexia-like group (*p* < 0.001). Similarly, receipt of systemic therapy (27.3% vs. 64.3%, *p* < 0.001) and radiotherapy (13.6% vs. 35.7%, *p* = 0.006) increased stepwise across phenotypes. Rates of local recurrence (15.9% vs. 42.9%, *p* = 0.003) and metastatic progression (11.4% vs. 35.7%, *p* = 0.011) were also progressively higher from muscle-preserved to cachexia-like patients.

### Identification and baseline characterization of AI-derived morphometric phenotypes

**Fig. 2:**
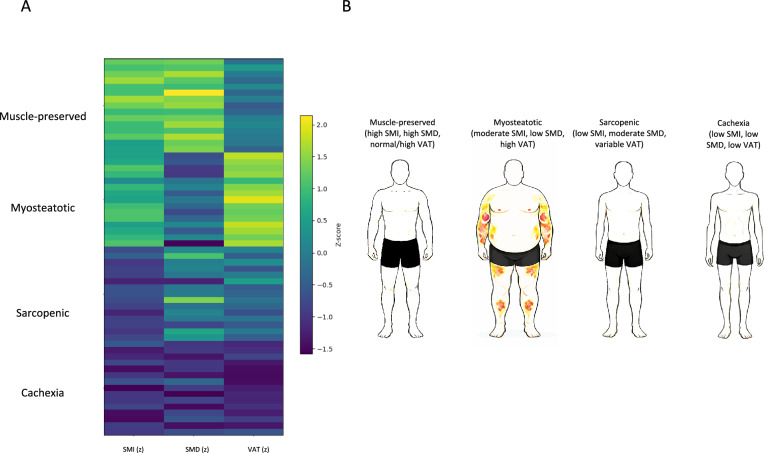
AI-based body composition clustering. **A** Example heat-map of AI-based clustering analysis with Skeletal muscle index, Visceral adipose tissue and skeletal muscle density. **B** Illustration of different body composition phenotypes (muscle-preserved, myosteatotic, sarcopenic, cachexia)

Unsupervised clustering of standardized preoperative SMI, SMD, and VAT identified four distinct morphometric phenotypes (Fig. 2A). Based on their multidimensional body composition profiles, these were interpreted as a muscle-preserved phenotype, a myosteatotic phenotype predominantly characterized by impaired muscle quality, a sarcopenic phenotype predominantly characterized by reduced muscle quantity, and a cachexia-like phenotype characterized by combined muscle depletion, low muscle attenuation, and reduced visceral adiposity (Fig. 2B).

Marked and systematic differences in morphometric parameters were observed between phenotypes. Patients assigned to the muscle-preserved phenotype demonstrated the highest skeletal muscle quantity and quality, with a mean SMI of 47.8 ± 6.3 cm^2^/m^2^ and a mean SMD of 41.6 ± 6.4 HU, accompanied by intermediate VAT levels (118 ± 56 cm^2^). This phenotype was considered representative of preserved muscle mass and favorable muscle composition.

The myosteatotic phenotype was characterized by disproportionately reduced muscle attenuation despite relatively preserved muscle quantity. Patients in this cluster showed a moderately reduced SMI (42.1 ± 5.9 cm^2^/m^2^) but markedly low SMD (28.9 ± 5.3 HU), indicating pronounced intramuscular fat infiltration. This phenotype also exhibited the highest visceral adiposity across all groups (VAT 142 ± 62 cm^2^), consistent with a myosteatotic, adiposity-dominant body composition profile.

The sarcopenic phenotype was primarily defined by low skeletal muscle quantity. These patients demonstrated a substantially reduced SMI (35.6 ± 4.8 cm^2^/m^2^) compared with muscle-preserved and myosteatotic patients, while muscle quality and adiposity showed intermediate values (SMD 34.0 ± 5.6 HU; VAT 96 ± 48 cm^2^). This phenotype therefore reflected a predominantly quantitative muscle-depleted profile without the pronounced fat infiltration seen in myosteatotic patients.

The cachexia-like phenotype represented the most unfavorable morphometric constellation. Patients in this cluster exhibited the lowest SMI (31.2 ± 4.4 cm^2^/m^2^), lowest SMD (26.4 ± 4.9 HU), and lowest VAT (64 ± 35 cm^2^), reflecting combined loss of muscle mass, severe deterioration of muscle quality, and depletion of visceral fat stores. This phenotype was interpreted as a cachexia-like body composition state.

Importantly, these phenotypes emerged exclusively from unsupervised clustering of CT-derived morphometric parameters and were generated independently of clinical characteristics or outcome data, supporting their data-driven and outcome-agnostic nature.

### Morphometric phenotypes are independently associated with overall survival and postoperative functional decline

To evaluate the prognostic and functional relevance of AI-derived body composition phenotypes in sarcoma surgery, we next analyzed their associations with OS and postoperative functional status.

**Fig. 3:**
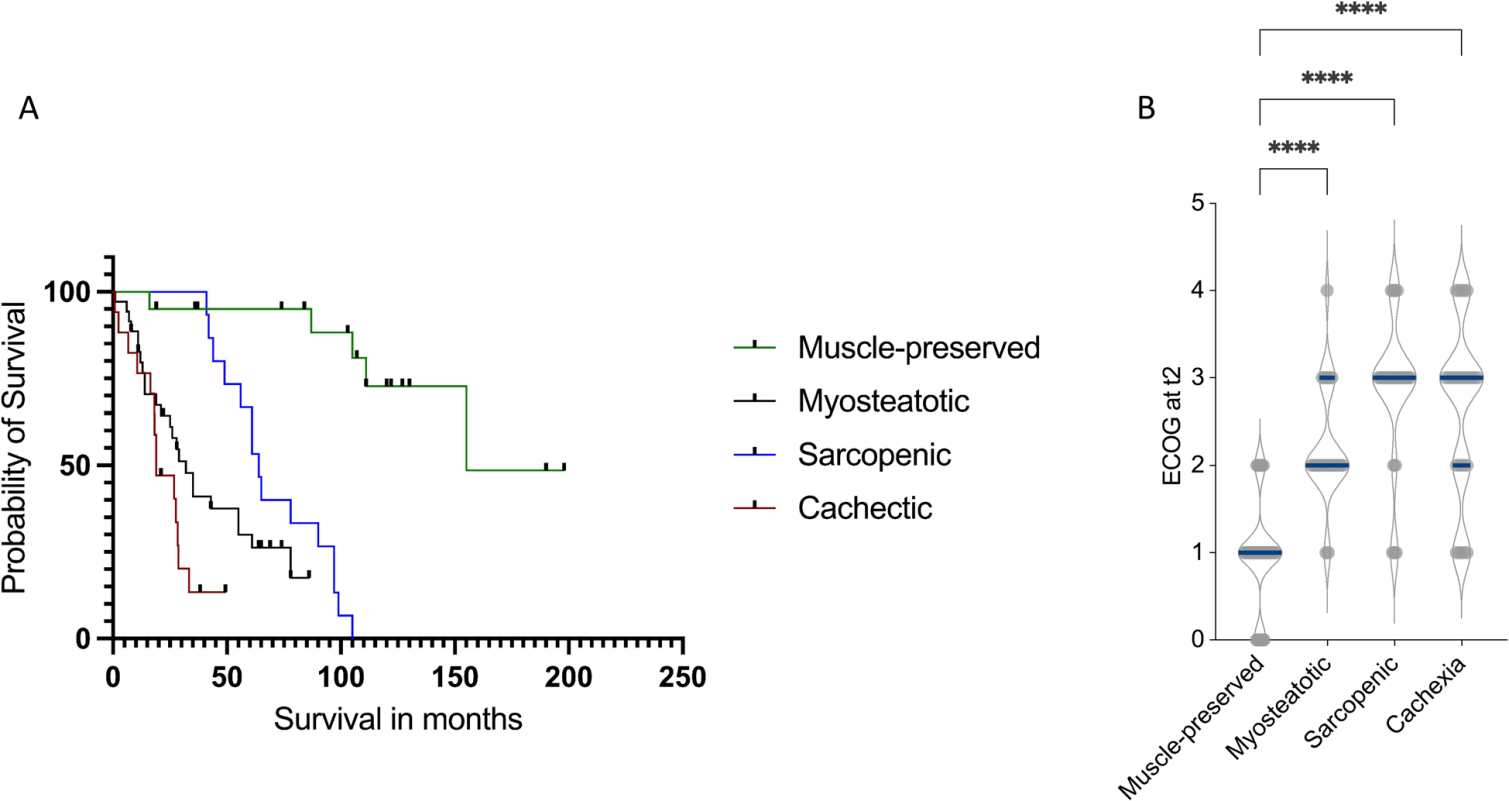
Comparison of survival and functional outcome in different AI-based body composition phenotypes. **A** Illustrated is the overall survival of patients with different body composition phenotypes (muscle-preserved, myosteatotic, sarcopenic, cachexia). Muscle-preserved patients show the best, cachectic patients the worst survival outcomes. **B** Functional outcomes differ by AI-based phenotypes as well, with cachectic and sarcopenic patients showing worst outcomes. Kruskal–Wallis Test has been applied. **** p < 0.0001

Kaplan–Meier analysis demonstrated pronounced and stepwise differences in OS across AI-derived morphometric phenotypes (Fig. 3A). Median OS was longest in muscle-preserved patients (155 months), followed by sarcopenic (64 months) and myosteatotic patients (32 months), and was shortest in cachexia-like patients (19 months; log-rank *p* < 0.0001).

Beyond survival, morphometric phenotypes were also strongly associated with postoperative functional status. ECOG performance status at follow-up differed significantly between phenotypes, demonstrating a progressive deterioration from muscle-preserved to cachexia-like patients (Fig. 3B). Median postoperative ECOG was 1 ± 0.5 in muscle-preserved patients, 2.5 ± 1 in myosteatotic patients, 3 ± 1 in sarcopenic patients, and 3 ± 0.5 in cachexia-like patients (*p* < 0.0001), indicating a substantial phenotype-dependent gradient of postoperative functional decline.

In multivariable Cox proportional hazards regression adjusting for age, preoperative ECOG performance status, Charlson Comorbidity Index, preoperative sarcopenia, sarcoma entity, local tumor recurrence, metastatic progression during follow-up, and lymph node involvement, morphometric phenotype remained independently associated with mortality (Table 2). Compared with the muscle-preserved phenotype, cachexia-like patients exhibited a more than three-fold increased risk of death (HR 3.28, 95% CI 2.01–5.36, *p* < 0.001), while sarcopenic patients also demonstrated significantly inferior survival (HR 1.89, 95% CI 1.26–2.83, *p* = 0.002). The myosteatotic phenotype showed a trend toward increased mortality risk that did not reach statistical significance after multivariable adjustment (HR 1.41, 95% CI 0.95–2.09, *p* = 0.081).

**Table 2:** Multivariable Cox regression for overall survival

Variable	HR	95% CI	*p*-value
Muscle-preserved phenotype (reference)	1.00	Reference	Reference
Myosteatotic phenotype	1.41	0.95–2.09	0.081
Sarcopenic phenotype	1.89	1.26–2.83	0.002
Cachexia-like phenotype	3.28	2.01–5.36	< 0.001
Preoperative sarcopenia	1.31	0.85–2.03	0.21
Age (per year)	1.04	0.88–1.06	0.43
ECOG performance status (preoperative)	1.28	1.08–1.90	0.008
Charlson Comorbidity Index	1.18	1.05–1.32	0.004
Local tumor recurrence	1.67	1.12–2.51	0.012
Metastatic progression during follow-up	1.85	1.07–3.19	0.026
Lymph node involvement	1.19	0.51–2.78	0.69
Sarcoma entity	1.16	0.72–1.87	0.53

Importantly, conventional preoperative sarcopenia was not independently associated with OS in the adjusted model (HR 1.31, 95% CI 0.85–2.03, *p* = 0.21). Conventional sarcopenia was defined using established sex-specific SMI cut-offs (< 52.4 cm^2^/m^2^ for males and < 38.5 cm^2^/m^2^ for females) [19, 22]. In contrast, preoperative ECOG performance status (HR 1.28, 95% CI 1.08–1.90, *p* = 0.008), Charlson Comorbidity Index (HR 1.18, 95% CI 1.05–1.32, *p* = 0.004), local tumor recurrence (HR 1.67, 95% CI 1.12–2.51, *p* = 0.012), and metastatic progression during follow-up (HR 1.85, 95% CI 1.07–3.19, *p* = 0.026) were identified as additional independent predictors of mortality.

### Morphometric phenotypes are independently associated with postoperative morbidity and healthcare utilization

To determine whether AI-derived morphometric phenotypes are associated with early postoperative morbidity, we next examined their relationships with SSI rates and length of hospital stay.

Postoperative SSI rates differed significantly across AI-derived morphometric phenotypes (*p* = 0.042). SSI occurred in 6.8% of muscle-preserved patients, 11.3% of myosteatotic patients, 14.3% of sarcopenic patients, and 21.4% of cachexia-like patients, demonstrating a stepwise increase in infectious complications across progressively unfavorable body composition phenotypes.

In multivariable Cox regression analysis adjusting for age, preoperative ECOG performance status, Charlson Comorbidity Index, preoperative sarcopenia, lymph node involvement, and sarcoma entity, morphometric phenotype remained independently associated with SSI risk (Table 3). Compared with muscle-preserved patients, cachexia-like patients exhibited a more than three-fold increased risk of postoperative SSI (HR 3.21, 95% CI 1.69–6.10, *p* < 0.001), while sarcopenic patients also demonstrated significantly elevated infection risk (HR 2.08, 95% CI 1.17–3.70, *p* = 0.012). The myosteatotic phenotype showed a non-significant trend toward higher SSI risk (HR 1.52, 95% CI 0.85–2.70, *p* = 0.16). Conventional preoperative sarcopenia was not independently associated with SSI in the adjusted model (HR 1.28, 95% CI 0.73–2.23, *p* = 0.39). Preoperative ECOG performance status (HR 1.34, *p* = 0.018) and Charlson Comorbidity Index (HR 1.12, *p* = 0.011) were identified as additional independent predictors of postoperative infection.

**Table 3:** Multivariable Cox regression for surgical site infection (SSI) rate

Variable	HR	95% CI	*p*-value
Muscle-preserved phenotype (reference)	1.00	Reference	Reference
Myosteatotic phenotype	1.52	0.85–2.70	0.16
Sarcopenic phenotype	2.08	1.17–3.70	0.012
Cachexia-like phenotype	3.21	1.69–6.10	< 0.001
Preoperative sarcopenia	1.28	0.73–2.23	0.39
Age (per year)	1.01	0.99–1.03	0.27
ECOG performance status (preoperative)	1.34	1.05–1.71	0.018
Charlson Comorbidity Index	1.12	1.03–1.23	0.011
Lymph node involvement	1.09	0.42–2.86	0.86
Sarcoma entity	1.06	0.78–1.46	0.71

Length of hospital stay increased progressively across morphometric phenotypes. In multivariable linear regression, sarcopenic and cachexia-like phenotypes were independently associated with prolonged hospitalization compared with muscle-preserved patients (Table 4). Sarcopenic patients stayed an average of 3.4 additional days (95% CI 1.3–5.5, *p* = 0.002), while cachexia-like patients required 6.2 additional days of hospitalization (95% CI 3.5–8.9, *p* < 0.001). The myosteatotic phenotype showed a non-significant trend toward longer LOS (+ 1.6 days, 95% CI − 0.3 to + 3.5, *p* = 0.10). Conventional preoperative sarcopenia was not independently associated with LOS (+ 0.8 days, *p* = 0.35). Increasing age, worse preoperative ECOG performance status, and higher Charlson Comorbidity Index were additional independent determinants of prolonged hospital stay.

**Table 4:** Multivariable linear regression for length of hospital stay (LOS)

Variable	β (days)	95% CI	*p*-value
Muscle-preserved	0.0	Reference	Reference
Myosteatotic phenotype	+ 1.6	− 0.3 to + 3.5	0.10
Sarcopenic phenotype	+ 3.4	+ 1.3 to + 5.5	0.002
Cachexia-like phenotype	+ 6.2	+ 3.5 to + 8.9	< 0.001
Preoperative sarcopenia	+ 0.8	− 0.9 to + 2.5	0.35
Age (per year)	+ 0.04	+ 0.01 to + 0.07	0.011
ECOG performance status (preoperative)	+ 1.5	+ 0.8 to + 2.2	< 0.001
Charlson Comorbidity Index	+ 0.6	+ 0.3 to + 0.9	< 0.001
Lymph node involvement	+ 0.5	− 1.8 to + 2.8	0.67
Sarcoma entity	+ 0.3	− 0.4 to + 1.0	0.41

To evaluate the potential impact of treatment-related alterations in body composition, a sensitivity analysis was performed after exclusion of all patients who received neoadjuvant systemic therapy. The analyzed cohort included 178 patients (56 patients excluded). In this restricted cohort, the associations between morphometric phenotypes and clinical outcomes remained directionally consistent. Adverse phenotypes continued to demonstrate significantly impaired outcomes across all endpoints. In multivariable Cox regression analysis, the cachexia-like phenotype remained strongly associated with reduced overall survival (HR 2.84, 95% CI 1.65–4.89, *p* < 0.001), while the sarcopenic phenotype also retained independent prognostic significance (HR 1.71, 95% CI 1.10–2.65, *p* = 0.018) (Supplementary Table 1). Similarly, both sarcopenic and cachexia-like phenotypes remained independently associated with increased risk of postoperative surgical site infection (HR 1.87, *p* = 0.043 and HR 2.89, *p* = 0.004, respectively; Supplementary Table 2). Length of hospital stay also remained significantly prolonged in sarcopenic and cachexia-like patients (+ 2.9 days, *p* =* p* = 0.005 and + 5.4 days, *p* < 0.001, respectively; Supplementary Table 3).

## Discussion

In this retrospective cohort study of patients undergoing curative-intent sarcoma resection, we demonstrate that AI-derived morphometric phenotypes based on routine preoperative CT imaging stratify patients into biologically and clinically distinct host profiles that are independently associated with OS, postoperative functional decline, SSI, and LOS. Using an unsupervised clustering approach integrating skeletal muscle quantity, muscle quality, and visceral adiposity, we identified four reproducible phenotypes (muscle-preserved, myosteatotic, sarcopenic, and cachexia-like) that captured a progressive gradient of host vulnerability. Importantly, these phenotypes provided prognostic and perioperative risk information beyond conventional sarcopenia and established clinical covariates.

The main finding of this study is the strong and stepwise association between morphometric phenotypes and OS. Cachexia-like and sarcopenic phenotypes exhibited markedly reduced survival, even after adjustment for age, comorbidity burden, performance status, sarcoma entity, and disease progression. In contrast, conventional CT-defined sarcopenia was not independently associated with mortality. This observation underscores the limitations of binary, single-parameter definitions and highlights the importance of multidimensional phenotyping. Patients with similar muscle quantity may differ substantially in muscle quality and adipose tissue reserves, reflecting distinct biological states ranging from isolated sarcopenia to advanced cancer-associated wasting. By integrating these dimensions, AI-derived phenotypes appear to better capture the systemic host response to malignancy and its relevance for long-term oncologic outcomes.

Beyond survival, morphometric phenotypes were strongly associated with postoperative functional status. Patients in unfavorable phenotypes demonstrated significantly worse ECOG performance status at follow-up, indicating that preoperative body composition is not only a prognostic marker but also relates closely to postoperative functional trajectories. This finding is particularly relevant in sarcoma care, where limb salvage, complex reconstructions, and prolonged rehabilitation place substantial demands on physiological reserve. Our results suggest that morphometric phenotyping may help identify patients at risk for postoperative functional decline, enabling earlier supportive interventions and more individualized perioperative counseling [3, 4, 27, 28].

Importantly, morphometric phenotypes were also independently associated with early postoperative morbidity. Cachexia-like and sarcopenic patients exhibited significantly higher SSI rates and substantially prolonged hospitalization. These findings align with growing evidence from oncologic and major surgical cohorts linking impaired muscle quality and depleted energy reserves to immune dysfunction, impaired wound healing, and reduced tolerance to surgical stress [7, 11, 12, 21]. The observation that conventional sarcopenia was not independently associated with SSI or LOS further supports the concept that adverse surgical outcomes are not driven by muscle mass alone, but rather by a complex interplay of muscle integrity, metabolic reserve, and systemic inflammation that is better reflected by integrated phenotypes.

Although data on CT-based body composition in sarcoma patients remain limited, our findings are consistent with and extend prior work in oncologic surgery. Previous studies have associated sarcopenia or myosteatosis with inferior survival and increased complications in soft tissue and bone sarcomas; however, these investigations largely relied on isolated parameters and predefined cut-offs [29, 30]. Our phenotype-based approach moves beyond these constraints by providing a data-driven framework that captures biological heterogeneity within and across traditional sarcopenia categories. Notably, the identification of a myosteatotic phenotype characterized by preserved muscle quantity but severely impaired muscle quality and high visceral adiposity emphasizes that “non-sarcopenic” patients are not necessarily metabolically or physiologically fit. The use of preoperative CT imaging reflects the patient’s physiological status at the time of surgical decision-making but may also incorporate treatment-related changes in body composition, particularly in patients receiving neoadjuvant chemotherapy. While baseline imaging at diagnosis could better isolate tumor-related effects, it would not capture the cumulative impact of disease and treatment on host physiology. Notably, sensitivity analyses excluding patients receiving neoadjuvant systemic therapy yielded consistent results, supporting the robustness of our findings.

From a clinical perspective, these findings have several important implications. All morphometric parameters used for phenotype generation are derived from routine preoperative CT scans that are already integral to sarcoma staging and surgical planning. AI-based morphometric phenotyping could therefore be readily integrated into existing workflows without additional imaging burden. Current sarcoma-specific prognostic tools such as the “Sarculator nomogram” primarily rely on tumor- and patient-related variables, including age, tumor size, histological subtype, and tumor grade, to estimate survival probabilities after surgical resection [31, 32]. While these models provide valuable oncologic risk stratification, they do not incorporate objective measures of host physiology or body composition. As a result, important dimensions of patient vulnerability, such as skeletal muscle depletion, impaired muscle quality, and altered adipose tissue reserves, remain unaccounted for. The present findings suggest that CT-based morphometric phenotyping captures a complementary host-related risk domain that is not reflected in existing sarcoma risk calculators.

Such phenotyping has the potential to augment current prognostic frameworks by incorporating an objective dimension of surgical risk. This may improve preoperative counseling, facilitate shared decision-making, and support individualized perioperative management strategies, including intensified nutritional support, prehabilitation programs, infection prevention protocols, and proactive postoperative care planning for high-risk phenotypes [33–35]. Importantly, morphometric phenotyping should not be viewed as a deterministic exclusion tool but rather as a means to contextualize surgical risk and tailor supportive interventions.

Several limitations must be acknowledged. First, the retrospective, single-center design introduces potential selection and information bias and limits generalizability. Second, the requirement for available preoperative CT imaging may have enriched the cohort for patients with more advanced disease or complex treatment courses. Third, although we adjusted for major clinical confounders, unmeasured factors such as inflammatory markers, detailed nutritional status, or socioeconomic variables could not be accounted for. Fourth, the sarcoma cohort comprised multiple histological subtypes, which may exhibit distinct biological behavior; while this reflects real-world sarcoma practice, subtype-specific analyses were underpowered. Neoadjuvant systemic therapy, particularly in osteosarcoma patients, may contribute to treatment-related muscle depletion and altered body composition. However, sensitivity analyses excluding these patients yielded consistent results, suggesting that the observed phenotype-outcome associations are not solely driven by treatment exposure. Finally, functional status was assessed using ECOG performance scores, which, although clinically relevant, provide only a coarse measure of postoperative function.

Future research should focus on external validation of morphometric phenotypes in multicenter sarcoma cohorts, prospective integration into clinical workflows, and investigation of dynamic body composition changes over the disease course. Combining morphometric phenotyping with laboratory biomarkers, molecular tumor profiles, and patient-reported outcomes may further refine risk stratification. Importantly, interventional studies are needed to determine whether targeted nutritional, metabolic, or exercise-based programs can favorably modify high-risk phenotypes and translate into improved surgical and oncologic outcomes.

In conclusion, AI-derived CT-based morphometric phenotyping identifies distinct host profiles in sarcoma patients undergoing curative resection and is independently associated with survival, postoperative functional decline, SSI, and LOS. These findings support the integration of multidimensional body composition analysis into sarcoma surgery as a clinically accessible tool to enhance prognostic assessment and personalize perioperative care.

## Supplementary Information

Below is the link to the electronic supplementary material.

Supplementary file1 (DOCX 13 kb)

## Declarations

### Data availability

Data can be made available upon request.

## References

[1] Sambri A, De Paolis M, Spinnato P, Donati DM, Bianchi G. The biology of myxofibrosarcoma: state of the art and future perspectives. Oncol Res Treat. 2020;43(6):314–22. 10.1159/000507334. 32450554 10.1159/000507334

[2] Strauss SJ, Frezza AM, Abecassis N, et al. Bone sarcomas: ESMO-EURACAN-GENTURIS-ERN PaedCan clinical practice guideline for diagnosis, treatment and follow-up. Ann Oncol. 2021;32(12):1520–36. 10.1016/j.annonc.2021.08.1995. 34500044 10.1016/j.annonc.2021.08.1995

[3] Lindner LH, Andreou D, Sebastian Bauer SB, et al. Osteosarcoma - S1 Guideline. 2023.

[4] Tlemsani C, Larousserie F, De Percin S, et al. Biology and management of high-grade chondrosarcoma: an update on targets and treatment options. Int J Mol Sci. 2023. 10.3390/ijms24021361. 36674874 10.3390/ijms24021361 PMC9862566

[5] Chow WA. Chondrosarcoma: biology, genetics, and epigenetics. F1000Res. 2018. 10.12688/f1000research.15953.1. 30519452 10.12688/f1000research.15953.1 PMC6248264

[6] Kattepur AK, Jones RL, Gulia A. Dedifferentiated chondrosarcoma: current standards of care. Future Oncol. 2021;17(35):4983–91. 10.2217/fon-2021-0830. 34734747 10.2217/fon-2021-0830

[7] Hosseini H, Heydari S, Hushmandi K, Daneshi S, Raesi R. Bone tumors: a systematic review of prevalence, risk determinants, and survival patterns. BMC Cancer. 2025;25(1):321. 10.1186/s12885-025-13720-0. 39984867 10.1186/s12885-025-13720-0 PMC11846205

[8] Hickey M, Farrokhyar F, Deheshi B, Turcotte R, Ghert M. A systematic review and meta-analysis of intralesional versus wide resection for intramedullary grade I chondrosarcoma of the extremities. Ann Surg Oncol. 2011;18(6):1705–9. 10.1245/s10434-010-1532-z. 21258968 10.1245/s10434-010-1532-z

[9] Nandakumar B, Baffour F, Abdallah NH, et al. Sarcopenia identified by computed tomography imaging using a deep learning-based segmentation approach impacts survival in patients with newly diagnosed multiple myeloma. Cancer. 2023;129(3):385–92. 10.1002/cncr.34545. 36413412 10.1002/cncr.34545 PMC9822865

[10] Zakaria HM, Elibe E, Macki M, et al. Morphometrics predicts overall survival in patients with multiple myeloma spine metastasis: a retrospective cohort study. Surg Neurol Int. 2018;9:172. 10.4103/sni.sni_383_17. 30210905 10.4103/sni.sni_383_17 PMC6122282

[11] Kylies J, Reiter A, Brauneck E, Striefler JK, Frosch KH, Priemel M. Prognostic and clinical implications of CT-morphometric sarcopenia in adult myxofibrosarcoma patients: a longitudinal analysis. World J Surg Oncol. 2025;23(1):403. 10.1186/s12957-025-04069-6. 41152884 10.1186/s12957-025-04069-6 PMC12570431

[12] Kylies J, Ballhause TM, Striefler J, Kylies D, Priemel M. The prognostic role of sarcopenia and muscle wasting in adult osteosarcoma patients: a longitudinal CT morphometric analysis. Eur J Surg Oncol. 2025;51(9):110293. 10.1016/j.ejso.2025.110293. 40627918 10.1016/j.ejso.2025.110293

[13] Sabel MS, Terjimanian M, Conlon AS, et al. Analytic morphometric assessment of patients undergoing colectomy for colon cancer. J Surg Oncol. 2013;108(3):169–75. 10.1002/jso.23366. 23846976 10.1002/jso.23366

[14] Yang XY, Hu Q. Association between sarcopenia and postoperative complications in patients undergoing surgery for gastrointestinal or hepato-pancreatico-biliary cancer. J Surg Oncol. 2023;128(4):708–10. 10.1002/jso.27371. 37355960 10.1002/jso.27371

[15] Romano A, Triarico S, Rinninella E, et al. Clinical impact of nutritional status and sarcopenia in pediatric patients with bone and soft tissue sarcomas: a pilot retrospective study (SarcoPed). Nutrients. 2022. 10.3390/nu14020383. 35057564 10.3390/nu14020383 PMC8781939

[16] Cao Q, Xiong Y, Zhong Z, Ye Q. Computed tomography-assessed sarcopenia indexes predict major complications following surgery for hepatopancreatobiliary malignancy: a meta-analysis. Ann Nutr Metab. 2019;74(1):24–34. 10.1159/000494887. 30513518 10.1159/000494887

[17] Whaikid P, Piaseu N. The effectiveness of protein supplementation combined with resistance exercise programs among community-dwelling older adults with sarcopenia: a systematic review and meta-analysis. Epidemiol Health. 2024;46:e2024030. 10.4178/epih.e2024030. 38374703 10.4178/epih.e2024030 PMC11369567

[18] Flexman AM, Street J, Charest-Morin R. The impact of frailty and sarcopenia on patient outcomes after complex spine surgery. Curr Opin Anaesthesiol. 2019;32(5):609–15. 10.1097/aco.0000000000000759. 31192792 10.1097/ACO.0000000000000759

[19] Brinkmann EJ, Wenger DE, Johnson JD, et al. Impact of preoperative sarcopenia in patients undergoing sacral tumor resection. J Surg Oncol. 2022;125(4):790–5. 10.1002/jso.26776. 34932215 10.1002/jso.26776

[20] Traeger L, Bedrikovetski S, Nguyen TM, et al. The impact of preoperative sarcopenia on postoperative ileus following colorectal cancer surgery. Tech Coloproctol. 2023;27(12):1265–74. 10.1007/s10151-023-02812-3. 37184771 10.1007/s10151-023-02812-3 PMC10638111

[21] Fujikawa H, Araki T, Okita Y, et al. Impact of sarcopenia on surgical site infection after restorative proctocolectomy for ulcerative colitis. Surg Today. 2017;47(1):92–8. 10.1007/s00595-016-1357-x. 27255541 10.1007/s00595-016-1357-x

[22] Bedrikovetski S, Traeger L, Jay AA, et al. Is preoperative sarcopenia associated with postoperative complications after pelvic exenteration surgery? Langenbecks Arch Surg. 2023;408(1):173. 10.1007/s00423-023-02913-5. 37133529 10.1007/s00423-023-02913-5 PMC10156810

[23] Chindapasirt J. Sarcopenia in cancer patients. Asian Pac J Cancer Prev. 2015;16(18):8075–7. 10.7314/apjcp.2015.16.18.8075. 26745041 10.7314/apjcp.2015.16.18.8075

[24] Abbod MF, Catto JW, Linkens DA, Hamdy FC. Application of artificial intelligence to the management of urological cancer. J Urol. 2007;178(4 Pt 1):1150–6. 10.1016/j.juro.2007.05.122. 17698099 10.1016/j.juro.2007.05.122

[25] Fusco R, Sansone M, Filice S, et al. Pattern recognition approaches for breast cancer DCE-MRI classification: a systematic review. J Med Biol Eng. 2016;36(4):449–59. 10.1007/s40846-016-0163-7. 27656117 10.1007/s40846-016-0163-7 PMC5016558

[26] Yassin NIR, Omran S, El Houby EMF, Allam H. Machine learning techniques for breast cancer computer aided diagnosis using different image modalities: a systematic review. Comput Methods Programs Biomed. 2018;156:25–45. 10.1016/j.cmpb.2017.12.012. 29428074 10.1016/j.cmpb.2017.12.012

[27] Nishio J, Nakayama S. Biology and management of high-grade myxofibrosarcoma: state of the art and future perspectives. Diagnostics. 2023. 10.3390/diagnostics13193022. 37835765 10.3390/diagnostics13193022 PMC10572210

[28] Vanni S, De Vita A, Gurrieri L, et al. Myxofibrosarcoma landscape: diagnostic pitfalls, clinical management and future perspectives. Ther Adv Med Oncol. 2022;14:17588359221093973. 10.1177/17588359221093973. 35782752 10.1177/17588359221093973 PMC9244941

[29] Albano D, Zanardo M, Basile M, et al. The impact of sarcopenia on sarcoma patients: a systematic review and meta-analysis. Radiol Med. 2025;130(9):1373–85. 10.1007/s11547-025-02016-9. 40459648 10.1007/s11547-025-02016-9 PMC12454457

[30] Phan EN, Thorpe SW, Wong FS, et al. Opportunistic muscle measurements on staging chest CT for extremity and truncal soft tissue sarcoma are associated with survival. J Surg Oncol. 2020;122(5):869–76. 10.1002/jso.26077. 32613648 10.1002/jso.26077 PMC8254594

[31] Borghi A, Gronchi A. Sarculator: how to improve further prognostication of all sarcomas. Curr Opin Oncol. 2024;36(4):253–62. 10.1097/cco.0000000000001051. 38726834 10.1097/CCO.0000000000001051

[32] Chowdhury A, Thway K, Pasquali S, et al. Opportunities and challenges in soft tissue sarcoma risk stratification in the era of personalised medicine. Curr Treat Options Oncol. 2024;25(8):1124–35. 10.1007/s11864-024-01244-x. 39080193 10.1007/s11864-024-01244-x

[33] Klein JD, Hey LA, Yu CS, et al. Perioperative nutrition and postoperative complications in patients undergoing spinal surgery. Spine (Phila Pa 1976). 1996;21(22):2676–82. 10.1097/00007632-199611150-00018. 8961455 10.1097/00007632-199611150-00018

[34] Hanna RM, Ghobry L, Wassef O, Rhee CM, Kalantar-Zadeh K. A practical approach to nutrition, protein-energy wasting, sarcopenia, and cachexia in patients with chronic kidney disease. Blood Purif. 2020;49(1–2):202–11. 10.1159/000504240. 31851983 10.1159/000504240

[35] Bossi P, Delrio P, Mascheroni A, Zanetti M. The spectrum of malnutrition/cachexia/sarcopenia in oncology according to different cancer types and settings: a narrative review. Nutrients. 2021. 10.3390/nu13061980. 34207529 10.3390/nu13061980 PMC8226689

