# Supplementary material for: AI-derived CT morphometric phenotypes predict survival, functional decline, and surgical morbidity following curative-intent surgical sarcoma resection
Source: J Orthop Surg Res. 2026 Apr 15;21:285. doi: 10.1186/s13018-026-06851-y (PMC13130809; doi:10.1186/s13018-026-06851-y)
Supplement: Supplementary file 1 — Supplementary file1 (DOCX 13 kb) [file 13018_2026_6851_MOESM1_ESM.docx]

**Supplementary Table 1: Multivariable Cox Regression for Overall Survival (Sensitivity Analysis)**

| **Variable** | **HR** | **95% CI** | **p-value** |
| --- | --- | --- | --- |
| **Muscle-preserved (reference)** | 1.00 | Reference | Reference |
| **Myosteatotic phenotype** | 1.32 | 0.87 – 2.01 | 0.19 |
| **Sarcopenic phenotype** | 1.71 | 1.10 – 2.65 | 0.018 |
| **Cachexia-like phenotype** | 2.84 | 1.65 – 4.89 | <0.001 |
| **Preoperative sarcopenia** | 1.22 | 0.78 – 1.91 | 0.38 |
| **Age (per year)** | 1.03 | 0.99 – 1.06 | 0.11 |
| **ECOG (preoperative)** | 1.25 | 1.05 – 1.75 | 0.012 |
| **Charlson Comorbidity Index** | 1.16 | 1.03 – 1.30 | 0.014 |
| **Local recurrence** | 1.59 | 1.04 – 2.42 | 0.032 |
| **Metastatic progression** | 1.74 | 1.01 – 3.00 | 0.046 |
| **Lymph node involvement** | 1.12 | 0.45 – 2.75 | 0.81 |
| **Sarcoma entity** | 1.14 | 0.69 – 1.88 | 0.61 |

**Supplementary Table 2 - Multivariable Cox Regression for Surgical Site Infection (SSI) Rate (Sensitivity Analysis)**

| **Variable** | **HR** | **95% CI** | **p-value** |
| --- | --- | --- | --- |
| **Muscle-preserved (reference)** | 1.00 | Reference | Reference |
| **Myosteatotic phenotype** | 1.41 | 0.75 – 2.66 | 0.28 |
| **Sarcopenic phenotype** | 1.87 | 1.02 – 3.43 | 0.043 |
| **Cachexia-like phenotype** | 2.89 | 1.41 – 5.92 | 0.004 |
| **Preoperative sarcopenia** | 1.21 | 0.66 – 2.21 | 0.54 |
| **Age (per year)** | 1.01 | 0.99 – 1.03 | 0.22 |
| **ECOG (preoperative)** | 1.29 | 1.02 – 1.63 | 0.031 |
| **Charlson Comorbidity Index** | 1.10 | 1.01 – 1.20 | 0.026 |
| **Lymph node involvement** | 1.05 | 0.39 – 2.81 | 0.92 |
| **Sarcoma entity** | 1.03 | 0.74 – 1.43 | 0.88 |

**Supplementary Table 3 - Multivariable Linear Regression for Length of Hospital Stay (Sensitivity Analysis)**

| **Variable** | **β (days)** | **95% CI** | **p-value** |
| --- | --- | --- | --- |
| **Muscle-preserved** | 0.0 | Reference | Reference |
| **Myosteatotic phenotype** | +1.3 | −0.5 to +3.1 | 0.15 |
| **Sarcopenic phenotype** | +2.9 | +0.9 to +4.9 | 0.005 |
| **Cachexia-like phenotype** | +5.4 | +2.8 to +8.0 | <0.001 |
| **Preoperative sarcopenia** | +0.6 | −1.1 to +2.3 | 0.48 |
| **Age (per year)** | +0.03 | +0.01 to +0.06 | 0.017 |
| **ECOG (preoperative)** | +1.3 | +0.7 to +1.9 | <0.001 |
| **Charlson Comorbidity Index** | +0.5 | +0.2 to +0.8 | 0.002 |
| **Lymph node involvement** | +0.4 | −1.7 to +2.5 | 0.71 |
| **Sarcoma entity** | +0.2 | −0.5 to +0.9 | 0.58 |
